# Prevalence and Correlates of Nomophobia and Insomnia Among Health Sciences Students: Evidence From a Cross‐Sectional Study

**DOI:** 10.1002/puh2.70419

**Published:** 2026-10-14

**Authors:** Nuha Fatima, Mohammed Waqqas, Husna Irfan Thalib, Nadeem Ikram, Sariya Khan, Samratul Fuadah, Mohammed Talha Mohammed Zubair, Zain Ul Abideen Nadeem, Mable Pereira

**Affiliations:** ^1^ General Medicine Practice Program Batterjee Medical College Jeddah Saudi Arabia; ^2^ College of Medicine Alfaisal University Riyadh Saudi Arabia; ^3^ School of Medicine Lincoln American University Georgetown Guyana

**Keywords:** cross‐sectional study, digital dependency, insomnia, nomophobia, sleep disturbance, smartphone addiction

## Abstract

**Background:**

Nomophobia is a behavioral condition characterized by fear or anxiety resulting from inability to access mobile phones. Excessive smartphone use has been associated with psychological and sleep‐related disturbances. University students, particularly health sciences students, may be especially vulnerable due to academic stress and high digital exposure.

**Objectives:**

This study aimed to determine the prevalence of nomophobia and insomnia symptoms among health sciences students and to assess the association between nomophobia severity and insomnia symptom severity.

**Methodology:**

A cross‐sectional analytical study was conducted among undergraduate health sciences students at Batterjee Medical College (BMC), Jeddah, Saudi Arabia. Data were collected using a structured electronic questionnaire including sociodemographic variables, the Nomophobia Questionnaire (NMP‐*Q*), and the Insomnia Severity Index (ISI). A total of 702 participants were included. Descriptive statistics, chi‐square test, Spearman's rank correlation, and binary logistic regression analysis were performed.

**Results:**

The mean nomophobia score was 77.21 ± 31.7. Overall, 96.6% of participants demonstrated nomophobia, with moderate nomophobia being the most prevalent category (45.3%), followed by severe nomophobia (26.1%). The mean insomnia score was 10.86 ± 5.7, and 71.4% of participants exhibited elevated insomnia symptoms, predominantly at the subthreshold level (46.3%). A statistically significant association was observed between nomophobia and insomnia symptoms (*p* < 0.001). Using continuous total scores, a weak positive correlation was identified between nomophobia severity and insomnia symptom severity (Spearman's *ρ* = 0.290, *p* < 0.001). Female gender was significantly associated with higher nomophobia prevalence (*p* = 0.014). In multivariable logistic regression analysis treating nomophobia as a continuous predictor, each 10‐point increase in NMP‐*Q* score was associated with significantly higher adjusted odds of insomnia symptoms (adjusted odds ratio [AOR] = 1.20, 95% confidence interval [CI] 1.13–1.27, *p* < 0.001) after adjustment for sociodemographic factors.

**Conclusion:**

Nomophobia and insomnia symptoms are highly prevalent among health sciences students. A weak but statistically significant association was observed; however, the cross‐sectional design and convenience sampling warrant cautious interpretation. These findings highlight the need for interventions promoting healthy smartphone use and sleep hygiene.

## Graphical abstract

Nomophobia and insomnia are highly prevalent among health sciences students, with a significant positive association between their severities. These findings emphasize the importance of promoting healthy smartphone use and sleep hygiene interventions in university populations.

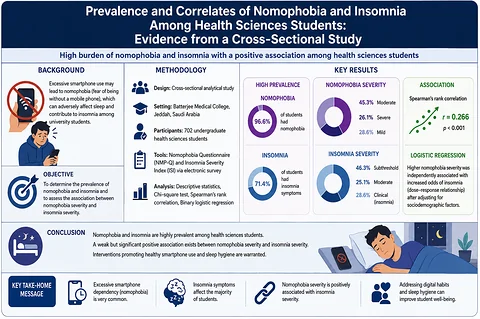

## 1 Introduction

Mobile phones have become an indispensable component of modern‐day life, especially for college students, whose daily routines rely heavily on mobile phones for communication and other activities. Nevertheless, the rising trend of utilizing smartphones for daily routines has led to various issues pertaining to the adverse effects of smartphone addiction leading to nomophobia amongst individuals. Nomophobia is referred to as the fear and anxiety experienced by individuals when they are deprived of access to their mobile phones [1].

Nomophobia can be considered a behavioral issue that also carries adverse consequences for individuals’ psychological well‐being. Research indicates that the rising trend of smartphone use has had an adverse effect on the psychological well‐being of individuals, including stress, depression, and low grades [2, 3]. College students are at particularly high risk of experiencing nomophobia, as their daily routines depend heavily on mobile phones, especially smartphones.

An emerging public health problem among university students is the disruption of sleep and insomnia. Sleep is one of the key elements affecting cognitive and psychological functioning and is also crucial for human health and well‐being. Recent changes in lifestyle, including smartphone use and participation in nighttime activities that require smartphones, have contributed to this problem. Blue light emitted by smartphones has been shown to cause negative effects on sleep quality and the circadian rhythm, including suppression of melatonin [4, 5].

Previous studies have reported associations between problematic smartphone use, nomophobia, and sleep disturbances among university students. For example, people with higher levels of smartphone addiction have been shown to have poor sleep quality, shorter sleep duration, and insomnia symptoms [6]. Among medical and other university students, the combination of academic stress and smartphone use is known to negatively affect sleep quality.

Medical students represent another population particularly vulnerable to nomophobia and insomnia, given their demanding academic schedules, irregular sleep patterns, and heavy reliance on technology for both academic and personal purposes. Numerous studies conducted worldwide have documented a high prevalence of nomophobia among medical students, with some reporting rates exceeding 80%. Medical students have similarly been shown to be at considerable risk of insomnia [7, 8, 9].

Several theoretical frameworks provide plausible mechanisms linking nomophobia with insomnia. Hyperarousal theory proposes that heightened cognitive and physiological arousal interferes with the initiation and maintenance of sleep. Individuals experiencing nomophobia may exhibit anticipatory anxiety regarding potential loss of connectivity, resulting in repetitive smartphone checking behaviors and persistent cognitive activation at bedtime. Similarly, the cognitive‐behavioral model of insomnia suggests that excessive pre‐sleep cognitive engagement and conditioned arousal contribute to sleep disturbances. The fear of missing out (FoMO) framework further explains that concerns about missing social interactions or information may encourage compulsive nighttime smartphone use, delaying sleep onset. From a behavioral addiction perspective, repetitive smartphone checking functions as a reinforcing behavior that becomes increasingly difficult to control, thereby perpetuating sleep disruption. Collectively, these frameworks support the hypothesis that nomophobia may be associated with insomnia through psychological and behavioral pathways rather than merely through smartphone ownership or use itself [1, 2, 3, 4, 5, 7, 8, 9].

Although numerous studies have examined smartphone dependence syndrome and insomnia separately, comparatively little research has addressed the relationship between nomophobia and insomnia specifically among Saudi populations. Clarifying this relationship is important for informing intervention strategies that promote healthy technology use among medical and health sciences students.

Therefore, this study assessed the relationship between nomophobia and insomnia symptoms among health sciences students at Batterjee Medical College (BMC), Jeddah, Saudi Arabia.

## 2 Methods

### 2.1 Study Design and Setting

This study employed a cross‐sectional analytical design and was conducted over a period of 6 months from January 2025 to July 2025 among undergraduate health sciences students enrolled at BMC, Jeddah, Saudi Arabia. The cross‐sectional design was selected to assess the prevalence of nomophobia and insomnia symptoms and to evaluate their association within the study population at a single point in time.

### 2.2 Sampling Technique

A non‐probability convenience sampling technique was used to recruit participants. Students who were available and willing to participate during the study period were invited to complete the online questionnaire. This sampling method was selected on the basis of feasibility, accessibility, and the defined study timeline.

### 2.3 Study Population

The study population consisted of undergraduate medical and health sciences students from all academic years.

**Inclusion criteria**:

- Undergraduate medical and health sciences students enrolled at BMC, Jeddah, Saudi Arabia
- Students from all academic levels
- Students who owned smartphones

**Exclusion criteria**:

- Students previously diagnosed with psychological disorders
- Students currently receiving pharmacological treatment for behavioral or psychological conditions. Psychological disorders and current pharmacological treatment status were identified on the basis of participant self‐reporting in the questionnaire.

These criteria were applied to reduce potential confounding factors affecting sleep quality or anxiety‐related behaviors.

### 2.4 Sample Size Determination

The minimum required sample size was calculated as 242 participants using the Raosoft online sample size calculator. The calculation was based on a confidence level of 95%, an assumed prevalence of 50%, and a margin of error of 5%.

### 2.5 Data Collection Procedure

Data were collected using a validated, self‐administered structured questionnaire distributed electronically via Microsoft Forms. The online format improved accessibility and minimized missing responses through structured mandatory fields. A total of 750 students were invited to participate in the study through class announcements and student WhatsApp messaging groups. Of these, 702 students completed the survey, yielding a response rate of 93.6%. No responses were excluded due to incomplete data (0/702, 0%). To minimize the possibility of duplicate submissions, Microsoft Forms was configured to allow only a single response per participant.

Participants were provided with study information prior to participation, and electronic informed consent was obtained before questionnaire access was granted.

The questionnaire consisted of three main sections:

1. Sociodemographic characteristics
2. Nomophobia Questionnaire (NMP‐*Q*)
3. Insomnia Severity Index (ISI)

### 2.6 Study Instruments

The NMP‐*Q*, developed by Yildirim et al., consists of 20 items rated on a 7‐point Likert scale ranging from 1 (strongly disagree) to 7 (strongly agree). Total scores range from 20 to 140 and are interpreted as follows: 20 indicates absence of nomophobia, 21–59 mild nomophobia, 60–99 moderate nomophobia, and 100–140 severe nomophobia. The instrument assesses four domains: inability to communicate, loss of connectedness, inability to access information, and giving up convenience.

The ISI is a validated 7‐item self‐report questionnaire assessing sleep difficulties over the past 2 weeks. Each item is scored from 0 to 4, with total scores ranging from 0 to 28. Scores are interpreted as 0–7 no clinically significant insomnia, 8–14 subthreshold insomnia, 15–21 moderate clinical insomnia, and 22–28 severe clinical insomnia.

The internal consistency of the NMP‐*Q* and ISI scales in the current study was assessed using Cronbach's alpha coefficient. The NMP‐*Q* and ISI demonstrated excellent internal consistency in the present study, with Cronbach's alpha coefficients of 0.93 and 0.86, respectively. Internal consistency alone does not establish construct validity. Both the NMP‐*Q* and ISI were administered in their original English versions. English is the language of instruction at BMC, and students receive their academic and clinical education in English. Therefore, the English versions were considered appropriate for administration to the study participants, and no translation or back‐translation was performed.

### 2.7 Data Management

Data collected from Microsoft Forms were exported to Microsoft Excel and subsequently transferred to Statistical Package for the Social Sciences (SPSS) software version 30 for analysis. Data cleaning was performed to remove incomplete responses and verify data consistency before statistical analysis.

### 2.8 Statistical Analysis

Statistical analysis was performed using SPSS software. Quantitative variables were summarized using mean and standard deviation. Qualitative variables were presented using frequencies and percentages. The chi‐square test was used to assess associations between categorical variables. Statistical significance was considered at *p* < 0.05.

For the binary logistic regression analysis, insomnia symptom status was dichotomized into absence of insomnia symptoms (ISI score 0–7) and presence of insomnia symptoms (ISI score ≥8), the latter including subthreshold, moderate, and severe insomnia symptoms. Because only 24 participants had no nomophobia (of whom 8 had insomnia symptoms), producing unstable odds ratios and wide confidence intervals (CIs) under a categorical parameterization, nomophobia severity (NMP‐*Q* total score) was instead modeled as a continuous predictor, expressed as the adjusted odds ratio (AOR) per 10‐point increase in NMP‐*Q* score; approximate linearity of this predictor in the logit was confirmed using the Box–Tidwell method (interaction *p* = 0.051). Independent variables entered into the model were age, gender, grade point average (GPA), nationality, academic level, academic discipline, and nomophobia severity. Reference categories were male gender, age <20 years, GPA 3.5–4.5, Saudi nationality, junior academic level, and medicine discipline. AORs with 95% CIs were calculated, and statistical significance was defined as *p* < 0.05. All 702 participants in the analytic sample had complete data on the variables entered into the model, so no missing‐data imputation was required. Multicollinearity among predictors was assessed using variance inflation factors (VIF); all VIFs were below 1.6, indicating no meaningful multicollinearity. Overall model fit was evaluated using the omnibus likelihood‐ratio test, Nagelkerke *R* ^2^, the Hosmer–Lemeshow goodness‐of‐fit test, and the model's classification accuracy and area under the receiver operating characteristic curve (AUC).

For regression analysis, junior students comprised Years 1–3, whereas senior students comprised Years 4–6. “Health sciences” included dentistry, pharmacy, nursing, respiratory therapy, radiologic sciences, physical therapy, occupational therapy, and healthcare administration, whereas medicine served as the reference category.

## 3 Results

A total of 702 participants were included in the study (Table 1). The majority of subjects in this study were within the age bracket of 21–25 years (68.2%), followed by those aged less than 20 years (21.7%) and more than 26 years old (10.1%). The mean age of the subjects was 22.6 ± 3.9 years. Of the total participants, 57.7% were females, and 42.3% were males. The majority of the subjects were singles (90.0%) and had Saudi nationality (66.4%). Regarding their academic status, 54.8% were among those having GPAs from 3.5 to 4.5. All academic years had students, but the second‐year and first‐year students made up the highest proportion, 21.9% and 21.5%, respectively. Medicine was the most numerous subject in terms of academics (38.9%). Mean nomophobia score was 77.21 ± 31.7, and moderate (45.3%) and severe (26.1%) levels of nomophobia existed among students. For insomnia symptoms, the mean score was 10.86 ± 5.7, and 71.4% of participants exhibited insomnia symptoms (ISI ≥ 8), predominantly at the subthreshold level (46.3%) (Table 1).

**TABLE 1:** Sociodemographic characteristics, nomophobia levels, and insomnia severity among study participants (*N* = 702).

Variable		*N*	%
**Age**	<20	152	21.7
	21–25	479	68.2
	>26	71	10.1
	Mean ± SD	22.6 ± 3.9	
**Gender**	Male	297	42.3
	Female	405	57.7
**Marital status**	Single	632	90.0
	Married	70	10.0
**Nationality**	Saudi	466	66.4
	Non‐Saudi	236	33.6
**Grade point average (GPA)**	3.5–4.5	385	54.8
	<3.5	125	17.8
	>4.5	192	27.4
**Academic level**	First year	151	21.5
	Second year	154	21.9
	Third year	133	18.9
	Fourth year	128	18.2
	Fifth year	53	7.5
	Sixth year	83	11.8
**Disciplines**	Medicine	273	38.9
	Dentistry	76	10.8
	Pharmacy	84	12.0
	Physical therapy	20	2.8
	Healthcare administration	25	3.6
	Nursing	74	10.5
	Occupational therapy	11	1.6
	Radiologic sciences	48	6.8
	Respiratory therapy	91	13.0
**Nomophobia**	Mean ± SD	77.21 ± 31.7	
	Absent (score = 20)	24	3.4
	Mild (21–59)	177	25.2
	Moderate (60–99)	318	45.3
	Severe (100–140)	183	26.1
	Any nomophobia (≥21)	678	96.6
**Insomnia**	Mean ± SD	10.86 ± 5.7	
	Absent (0–7)	201	28.6
	Subthreshold (8–14)	325	46.3
	Moderate insomnia (15–21)	145	20.7
	Severe insomnia (22–28)	31	4.4
	Any insomnia (≥8)	501	71.4

The relationship between sociodemographic factors and the level of nomophobia was investigated (Table 2). None of the sociodemographic factors, except for gender, correlated significantly with nomophobia levels, including age, marital status, nationality, GPA, academic level, or academic discipline. Gender had a statistically significant relationship with the overall presence of nomophobia (*p* = 0.014). Despite variations in the distribution of nomophobia levels by sociodemographic characteristics, most subjects were found to have moderate to high levels of nomophobia.

**TABLE 2:** Association between sociodemographic characteristics and nomophobia severity among participants.

Variable	Category	No nomophobia (NMP‐*Q* = 20), *n* (%) (total *n* = 24)	Mild (21–59), *n* (%) (total *n* = 177)	Moderate (60–99), *n* (%) (total *n* = 318)	Severe (100–140), *n* (%) (total *n* = 183)	*p* value	Any nomophobia (≥21), *n* (%) (total *n* = 678)	*p* value
Age	<20	7 (29%)	36 (20%)	82 (26%)	27 (15%)	0.67	145 (21%)	0.298
	21–25	13 (54%)	124 (70%)	203 (64%)	139 (76%)		466 (69%)	
	>26	4 (17%)	17 (10%)	33 (10%)	17 (9%)		67 (10%)	
Gender	Male	16 (67%)	68 (38%)	137 (43%)	76 (42%)	0.07	281 (41%)	0.014^*^
	Female	8 (33%)	109 (62%)	181 (57%)	107 (58%)		397 (59%)	
Marital status	Single	22 (92%)	162 (92%)	289 (91%)	159 (87%)	0.426	610 (90%)	0.785
	Married	2 (8%)	15 (8%)	29 (9%)	24 (13%)		68 (10%)	
Nationality	Saudi	16 (67%)	108 (61%)	223 (70%)	119 (65%)	0.218	450 (66%)	0.976
	Non‐Saudi	8 (33%)	69 (39%)	95 (30%)	64 (35%)		228 (34%)	
Grade point average (GPA)	3.5–4.5	10 (42%)	87 (49%)	188 (59%)	100 (55%)	0.226	375 (55%)	0.396
	<3.5	5 (21%)	31 (18%)	56 (18%)	33 (18%)		120 (18%)	
	>4.5	9 (38%)	59 (33%)	74 (23%)	50 (27%)		183 (26.9%)	
Academic level	First year	6 (25%)	35 (20%)	76 (24%)	34 (19%)	0.826	145 (21%)	0.868
	Second year	7 (29%)	34 (19%)	75 (24%)	38 (21%)		147 (22%)	
	Third year	5 (21%)	37 (21%)	57 (18%)	34 (19%)		128 (19%)	
	Fourth year	3 (13%)	37 (21%)	51 (16%)	37 (20%)		125 (18%)	
	Fifth year	1 (4%)	13 (7%)	20 (6%)	19 (10%)		52 (8%)	
	Sixth year	2 (8%)	21 (12%)	39 (12%)	21 (11%)		81 (12%)	
Discipline	Medicine	9 (38%)	82 (46%)	114 (36%)	68 (37%)	0.204	264 (39%)	0.771
	Dentistry	1 (4%)	12 (7%)	34 (11%)	29 (16%)		75 (11%)	
	Pharmacy	2 (8%)	13 (7%)	47 (15%)	22 (12%)		82 (12%)	
	Physical therapy	0 (0%)	9 (5%)	8 (3%)	3 (2%)		20 (3%)	
	Healthcare admin	1 (4%)	5 (3%)	13 (4%)	6 (3%)		24 (4%)	
	Nursing	3 (13%)	21 (12%)	29 (9%)	21 (11%)		71 (10%)	
	Occupational therapy	0 (0%)	1 (1%)	7 (2%)	3 (2%)		11 (2%)	
	Radiologic sciences	3 (13%)	13 (7%)	21 (7%)	11 (6%)		45 (7%)	
	Respiratory therapy	5 (21%)	21 (12%)	45 (14%)	20 (11%)		86 (13%)	

*Note:* Percentages are column percentages, calculated within each nomophobia severity category (i.e., the denominator for each percentage is the column total shown in the header).

Abbreviation: NMP‐*Q*, Nomophobia Questionnaire.

^*^Indicates significant *p* value.

Associations between sociodemographic variables and severity of insomnia symptoms were assessed (see Table 3). Insomnia symptom severity did not correlate significantly with any sociodemographic variables, including age, sex, marital status, nationality, GPA, or academic year. Academic discipline correlated significantly with severity of insomnia symptoms (*p* = 0.003) and with the presence of any insomnia symptoms (*p* = 0.017). Insomnia symptoms, including subthreshold insomnia symptoms, were identified in all sociodemographic groups studied, with subthreshold insomnia symptoms being the most frequent category.

**TABLE 3:** Association between sociodemographic characteristics and insomnia severity among participants.

Variable	Category	No clinically significant insomnia (ISI 0–7), *n* (%) (total *n* = 201)	Subthreshold insomnia (ISI 8–14), *n* (%) (total *n* = 325)	Moderate clinical insomnia (ISI 15–21), *n* (%) (total *n* = 145)	Severe clinical insomnia (ISI 22–28), *n* (%) (total *n* = 31)	*p* value	Any insomnia (ISI ≥8), *n* (%) (total *n* = 501)	*p* value
**Age**	<20	41 (20%)	73 (22%)	33 (23%)	5 (16%)	0.489	111 (22%)	0.104
	21–25	132 (66%)	223 (69%)	101 (70%)	23 (74%)		347 (69%)	
	>26	28 (14%)	29 (9%)	11 (8%)	3 (10%)		43 (9%)	
**Gender**	Male	91 (45%)	138 (42%)	57 (39%)	11 (35%)	0.604	206 (41%)	0.314
	Female	110 (55%)	187 (58%)	88 (61%)	20 (65%)		295 (59%)	
**Marital status**	Single	181 (90%)	287 (88%)	135 (93%)	29 (94%)	0.387	451 (90%)	0.99
	Married	20 (10%)	38 (12%)	10 (7%)	2 (6%)		50 (10%)	
**Nationality**	Saudi	127 (63%)	218 (67%)	103 (71%)	18 (58%)	0.34	339 (68%)	0.256
	Non‐Saudi	74 (37%)	107 (33%)	42 (29%)	13 (42%)		162 (32%)	
**Grade point average (GPA)**	3.5–4.5	99 (49%)	196 (60%)	70 (48%)	20 (65%)	0.055	286 (57%)	0.07
	<3.5	35 (17%)	52 (16%)	33 (23%)	5 (16%)		90 (18%)	
	>4.5	67 (33%)	77 (24%)	42 (29%)	6 (19%)		125 (25%)	
**Academic level**	First year	44 (22%)	70 (22%)	31 (21%)	6 (19%)	0.25	107 (21%)	0.668
	Second year	44 (22%)	76 (23%)	25 (17%)	9 (29%)		110 (22%)	
	Third year	39 (19%)	65 (20%)	26 (18%)	3 (10%)		94 (19%)	
	Fourth year	32 (16%)	64 (20%)	29 (20%)	3 (10%)		96 (19%)	
	Fifth year	20 (10%)	20 (6%)	11 (8%)	2 (6%)		33 (7%)	
	Sixth year	22 (11%)	30 (9%)	23 (16%)	8 (26%)		61 (12%)	
**Discipline**	Medicine	87 (43%)	116 (36%)	57 (39%)	13 (42%)	0.003	186 (37%)	0.017
	Dentistry	10 (5%)	42 (13%)	21 (14%)	3 (10%)		66 (13%)	
	Pharmacy	27 (13%)	26 (8%)	21 (14%)	10 (32%)		57 (11%)	
	Physical therapy	3 (1%)	13 (4%)	4 (3%)	0 (0%)		17 (3%)	
	Healthcare admin	4 (2%)	14 (4%)	7 (5%)	0 (0%)		21 (4%)	
	Nursing	19 (9%)	37 (11%)	16 (11%)	2 (6%)		55 (11%)	
	Occupational therapy	2 (1%)	7 (2%)	2 (1%)	0 (0%)		9 (2%)	
	Radiologic sciences	16 (8%)	23 (7%)	8 (6%)	1 (3%)		32 (6%)	
	Respiratory therapy	33 (16%)	47 (14%)	9 (6%)	2 (6%)		58 (12%)	

*Note:* Percentages are column percentages, calculated within each insomnia symptom severity category (i.e., the denominator for each percentage is the column total shown in the header).

Abbreviation: ISI, Insomnia Severity Index.

A significant association was found between nomophobia and insomnia symptom levels (*p* < 0.001) (Table 4), based on chi‐square analysis. Individuals who reported experiencing high‐level nomophobia were characterized by a higher probability of exhibiting insomnia symptoms. The highest prevalence of severe insomnia symptoms was recorded in individuals with severe nomophobia (61%). Moreover, the incidence of moderate and subthreshold insomnia symptoms was greater in individuals with mild/moderate and severe nomophobia than those with no nomophobia. In general, participants with higher levels of nomophobia demonstrated a greater proportion of insomnia symptoms, with severe insomnia symptoms being most frequent among those with severe nomophobia.

**TABLE 4:** Association between nomophobia severity and insomnia levels among participants.

Nomophobia level	No clinically significant insomnia (ISI 0–7), *n* (%) (total *n* = 201)	Subthreshold insomnia (ISI 8–14), *n* (%) (total *n* = 325)	Moderate clinical insomnia (ISI 15–21), *n* (%) (total *n* = 145)	Severe clinical insomnia (ISI 22–28), *n* (%) (total *n* = 31)	Any insomnia (≥8) (*n* = 501)	*p* value
No nomophobia (score = 20)	16 (8.0%)	5 (2.0%)	2 (1.4%)	1 (3.2%)	8 (1.6%)	<0.001
Mild nomophobia (score 21–59)	77 (38.3%)	71 (21.8%)	26 (17.9%)	3 (9.7%)	100 (20.1%)	
Moderate nomophobia (score 60–99)	77 (38.3%)	164 (50.5%)	69 (47.6%)	8 (25.8%)	241 (48.9%)	
Severe nomophobia (score 100–140)	31 (15.4%)	85 (26.2%)	48 (33.1%)	19 (61.3%)	152 (30.8%)	

*Note:* Percentages are column percentages, calculated within each insomnia symptom severity category (i.e., the denominator for each percentage is the column total shown in the header), not within each nomophobia level.

Abbreviation: ISI, Insomnia Severity Index.

Because the categorical Spearman correlation reported in an earlier stage of this analysis used ordinal NMP‐*Q* and ISI severity grades rather than total scores, the association was re‐examined using continuous total scores (Table 5, Figure 1). A Spearman rank correlation between the continuous NMP‐*Q* total score and the continuous ISI total score showed a significant positive correlation (*ρ* = 0.290, *p* < 0.001, *N* = 702), consistent with the correlation obtained using ordinal severity categories (*ρ* = 0.266, *p* < 0.001). These findings demonstrate a statistically significant but modest positive association between nomophobia severity and insomnia symptom severity. The magnitude of this correlation indicates that nomophobia explains only a limited proportion of variability in insomnia symptoms, suggesting that sleep disturbance is likely influenced by multiple interacting behavioral, psychological, and environmental factors.

**TABLE 5:** Spearman's rank correlation between continuous Nomophobia Questionnaire (NMP‐*Q*) total score and continuous Insomnia Severity Index (ISI) total score.

Variables		Nomophobia (NMP‐*Q* total score)	Insomnia (ISI total score)
Spearman's rho	Correlation coefficient (*ρ*)	1.000	0.290^**^
	Sig. (two‐tailed)	—	<0.001
	*N*	702	702
	Correlation coefficient (*ρ*)	0.290^**^	1.000
	Sig. (two‐tailed)	<0.001	—
	*N*	702	702

*Note:* Correlation is significant at the 0.01 level (two‐tailed), using continuous NMP‐*Q* and ISI total scores. For comparison, the correlation using ordinal NMP‐*Q*/ISI severity categories was *ρ* = 0.266 (*p* < 0.001). Statistical significance was set at *p* < 0.05. *N* = number of participants; *ρ* = Spearman's correlation coefficient.

^**^ The value of 0.266 represents the critical threshold for a correlation coefficient to reach statistical significance at the (*p* < 0.001) level given our sample size. Because the observed correlation of 0.290 exceeds this threshold, it is statistically significant at (*p* < 0.001).

**FIGURE 1:**
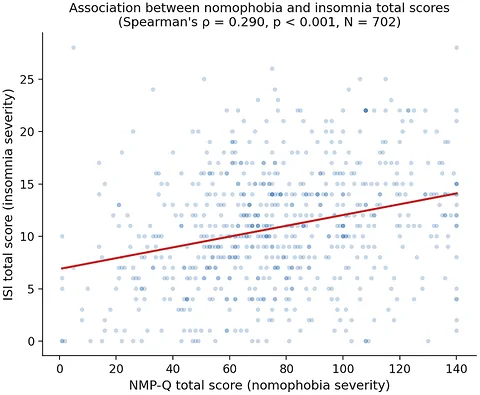
Scatterplot of the association between continuous NMP‐*Q* total score (nomophobia severity) and continuous ISI total score (insomnia symptom severity), with linear fit line (Spearman's *ρ* = 0.290, *p* < 0.001, *N* = 702). ISI, Insomnia Severity Index; NMP‐*Q*, Nomophobia Questionnaire.

### 3.1 Multivariable Analysis of Factors Associated With Insomnia

Binary logistic regression analysis was performed to identify factors independently associated with insomnia symptoms among health sciences students, with nomophobia modeled as a continuous predictor (per 10‐point increase in NMP‐*Q* score) rather than in exposure categories, as described in Section 2.8. After adjustment for age, gender, GPA, nationality, academic level, and academic discipline, each 10‐point increase in NMP‐*Q* score was associated with significantly higher adjusted odds of insomnia symptoms (AOR = 1.20, 95% CI 1.13–1.27, *p* < 0.001) (Table 6). Participants aged >26 years had significantly lower adjusted odds of insomnia symptoms than those aged <20 years (AOR = 0.48, 95% CI 0.25–0.94, *p* = 0.032); no other sociodemographic covariate was independently associated with insomnia symptoms after adjustment. The overall model was statistically significant (omnibus likelihood‐ratio *χ* ^2^ (9) = 55.28, *p* < 0.001) and showed acceptable discrimination (Nagelkerke *R* ^2^ = 0.109; AUC = 0.66), although the Hosmer–Lemeshow test indicated some deviation from perfect calibration (*χ* ^2^ (8) = 16.86, *p* = 0.032), which is discussed as a limitation.

**TABLE 6:** Multivariable binary logistic regression analysis of factors associated with insomnia symptoms (nomophobia modeled as a continuous predictor).

Variable	Category (reference)	Adjusted OR	95% CI	*p* value
Age	21–25 vs. <20	0.81	0.51–1.27	0.36
Age	>26 vs. <20	0.48	0.25–0.94	0.032^*^
Gender	Female vs. male	1.08	0.76–1.54	0.66
Grade point average (GPA)	<3.5 vs. 3.5–4.5	0.92	0.58–1.48	0.74
GPA	>4.5 vs. 3.5–4.5	0.70	0.47–1.05	0.083
Nationality	Non‐Saudi vs. Saudi	0.91	0.62–1.33	0.63
Academic level	Senior (Year 4–6) vs. junior (Year 1–3)	1.13	0.77–1.67	0.52
Discipline	Health sciences vs. medicine	1.17	0.81–1.68	0.40
Nomophobia (NMP‐*Q* score)	AOR per 10‐point increase	1.20	1.13–1.27	<0.001*

*Note:* Model diagnostics: Omnibus likelihood‐ratio *χ* ^2^ (9) = 55.28, *p* < 0.001; Nagelkerke *R* ^2^ = 0.109 (Cox–Snell *R* ^2^ = 0.076; McFadden *R* ^2^ = 0.066); Hosmer–Lemeshow goodness‐of‐fit *χ* ^2^ (8) = 16.86, *p* = 0.032; overall classification accuracy = 73.9% (sensitivity 96.4%, specificity 17.9% at a 0.5 cutoff); AUC = 0.66; *N* = 702 with no missing data on model variables; maximum VIF = 1.53, indicating no meaningful multicollinearity.

Abbreviations: AOR, adjusted odds ratio; CI, confidence interval; NMP‐*Q*, Nomophobia Questionnaire; VIF, variance inflation factor; vs., versus.

^*^ Astriek shows the *p* value > 0.05.

## 4 Discussion

This study aimed to determine the prevalence of nomophobia and insomnia symptoms among undergraduate health sciences students and to examine the association between these two conditions. The results of this study demonstrated a high prevalence of both nomophobia and insomnia symptoms, at 96.6% and 71.4%, respectively, and identified a statistically significant positive association between nomophobia severity and insomnia symptom severity. These findings suggest that smartphone‐related dependence may be associated with sleep disturbances among university students.

According to the findings of the present study, moderate nomophobia was the most common category, accounting for 45.3% of participants, followed by severe nomophobia, which accounted for 26.1%. These findings indicate that although the most extreme manifestations of nomophobia are not experienced by all students, a considerable proportion experience at least moderate levels of nomophobia.

The high prevalence of nomophobia observed in the current study is consistent with previous research conducted among university and medical students in different parts of the world. For example, research conducted among medical students in India found that over 90% of medical students experience some form of nomophobia, with moderate nomophobia being the most prevalent category [10]. Another study among medical students in India reported that approximately 83% suffered from moderate to severe forms of nomophobia, demonstrating the high level of smartphone dependence among students both in India and globally [3]. Similar findings were reported among university students in Turkey, where Yildirim et al. documented a high prevalence of nomophobia [11]. The high levels of nomophobia observed in the present study may reflect the increasing dependence on smartphones for academic, social, and daily activities among students in the modern era.

Gender differences were also observed, with female participants demonstrating higher levels of nomophobia than male participants. This finding is supported by previous studies reporting higher levels of nomophobia among women, potentially because females may rely more heavily on smartphones for social communication and interpersonal connectivity. For instance, Lee et al. reported that women demonstrated greater dependence on smartphone use because of its role in maintaining social connections [12]. Similarly, a study conducted among university students in Spain found higher levels of nomophobia among female participants, which was attributed to greater engagement in social networking activities [13].

Regarding sleep outcomes, most participants (71.4%) experienced some degree of insomnia symptoms, with subthreshold insomnia symptoms being the most prevalent category. This finding is consistent with previous studies reporting high rates of sleep disturbance among medical students. For example, the systematic review by Azad et al. demonstrated that medical students frequently experience poor sleep quality because of academic stressors, inconsistent sleep schedules, and increased use of electronic devices [14]. Likewise, Lund et al. reported that university students experienced sleep deprivation and elevated insomnia symptoms at higher rates than the general population [15].

A weak positive association was observed between nomophobia severity and insomnia symptom severity, suggesting a modest relationship between smartphone dependency and sleep disturbance. Demirci et al. similarly reported that smartphone addiction was significantly associated with poor sleep quality, daytime dysfunction, and depressive symptoms among university students [6]. Additionally, Exelmans and Van den Bulck found that excessive nighttime smartphone use was associated with reduced sleep duration and poorer sleep efficiency [5]. Although statistically significant, the magnitude of the observed association in the present study was modest, indicating that nomophobia likely represents only one of several factors associated with insomnia symptoms.

Several behavioral and psychological mechanisms have been proposed in the literature to explain associations between nomophobia and insomnia symptoms, although these mechanisms were not directly measured in the present study and should be interpreted as hypotheses for future research rather than as explanations established by these data. One proposed mechanism is that nighttime smartphone screen exposure may disrupt circadian rhythm regulation and sleep quality; exposure to smartphone screens has been shown elsewhere to suppress melatonin production, potentially delaying sleep onset and altering normal sleep patterns [4]. Excessive smartphone engagement has also been proposed to result in cognitive overstimulation that may make it more difficult for individuals to initiate sleep, although this pathway was not directly tested in the present study.

Psychological factors have also been proposed as potential contributors to this relationship. Individuals with greater tendencies toward smartphone dependence may experience higher levels of anxiety related to social interactions, FoMO, and continuous information seeking, which could theoretically increase cognitive and physiological arousal at bedtime and interfere with sleep initiation and maintenance. Przybylski et al. identified FoMO as an important factor driving nighttime smartphone use and negatively affecting sleep in other populations [16]; however, FoMO, anxiety, and related psychological constructs were not assessed in the present study, and this pathway therefore remains hypothetical in the context of the current findings.

The current study further demonstrated that most sociodemographic factors were not significantly associated with nomophobia severity, with the exception of gender. This finding is consistent with several previous studies suggesting that problematic smartphone‐related behaviors may occur across different academic levels and demographic groups. In contrast, academic discipline was significantly associated with insomnia symptom severity, which may reflect differences in academic workload and training demands across healthcare specialties.

The findings of this study have important practical implications because they suggest that problematic smartphone‐related behaviors may be associated with cognitive performance and mental well‐being. Previous research has shown that unhealthy sleep patterns among medical students may be negatively related to academic performance and burnout [17]. Therefore, interventions promoting healthier smartphone‐related behaviors and better sleep hygiene may represent useful strategies for improving student well‐being and academic functioning.

The multivariable binary logistic regression analysis further supported these findings by demonstrating that, after adjustment for age, gender, GPA, nationality, academic level, and academic discipline, each 10‐point increase in NMP‐*Q* score was associated with significantly higher adjusted odds of insomnia symptoms (AOR = 1.20, 95% CI 1.13–1.27, *p* < 0.001), consistent with a graded, dose–response‐like relationship between nomophobia severity and insomnia symptoms. Modeling nomophobia as a continuous predictor, rather than in categories, avoided the instability associated with the very small “no nomophobia” reference group (*n* = 24, of whom only 8 had insomnia symptoms) identified earlier, and a sensitivity check using the original nomophobia severity categories showed a broadly consistent, though less precise, pattern of increasing odds with increasing nomophobia severity. These findings suggest that the observed association is not explained solely by demographic or academic characteristics but remains evident after adjustment for these measured variables.

Several limitations should be considered when interpreting the findings of this study. First, the cross‐sectional design limits the ability to establish causal relationships between nomophobia and insomnia symptoms. Because exposure and outcome were measured simultaneously, temporal sequencing cannot be determined, and reverse causation remains possible. Consequently, causal inferences cannot be drawn from the present findings. Second, convenience sampling from a single institution may limit the generalizability of the findings and introduce selection bias. Third, the use of self‐reported questionnaires may lead to recall and reporting bias. Additionally, several important variables that may influence both nomophobia and insomnia symptoms, including caffeine consumption, total daily screen time, bedtime smartphone use, sleep hygiene practices, academic stress, symptoms of anxiety or depression, and physical activity, were not measured or controlled for. Consequently, residual confounding may partially explain the observed association. Finally, although the regression model was statistically significant overall and showed no meaningful multicollinearity among predictors, its discriminative ability was modest (AUC = 0.66), and the Hosmer–Lemeshow test indicated some imperfect calibration, indicating that unmeasured factors likely contribute substantially to insomnia symptom risk beyond nomophobia and the sociodemographic variables examined.

Future multicenter longitudinal studies incorporating behavioral, psychological, and lifestyle variables, together with comprehensive multivariable analyses, are recommended to better clarify the temporal relationship and underlying mechanisms linking nomophobia and insomnia among health sciences students.

## 5 Conclusion

This study demonstrated a high prevalence of nomophobia and insomnia symptoms among health sciences students. Although a statistically significant association was observed between nomophobia severity and insomnia symptom severity—evident both in continuous‐score correlation analysis and in a multivariable model treating nomophobia as a continuous predictor—the magnitude of this relationship was modest, suggesting that nomophobia represents only one of several factors associated with sleep disturbance. Given the cross‐sectional design, causal relationships cannot be inferred. Future longitudinal and behavioral studies incorporating additional psychological and lifestyle variables are warranted to better understand the mechanisms underlying this association and to inform targeted interventions.

## Author Contributions

All authors contributed to data collection, manuscript drafting, and revision and approved the final version of the manuscript.

## Funding

The authors have nothing to report.

## Ethics Statement

Ethical approval for this study was obtained from the Institutional Research and Ethics Committee of Batterjee Medical College, Jeddah, Saudi Arabia (Approval Code: RES‐2023‐0037). Participant recruitment and data collection were conducted from January to July 2025 under the approved study protocol. Participant anonymity and confidentiality were maintained throughout the study. Data were securely stored and were accessible only to the research team. Participation in the study was voluntary.

## Consent

Electronic informed consent was obtained from all participants prior to data collection.

## Conflicts of Interest

The authors declare no conflicts of interest.

## Data Availability

The datasets generated and analyzed during the current study are available from the corresponding author upon reasonable request.
